# Nurses’ knowledge of care of adult and pediatric patients tracheostomized patients: a cross-sectional study

**DOI:** 10.1186/s12912-025-03794-3

**Published:** 2025-08-30

**Authors:** Manuel Rodríguez–Darias, Candelaria de la Merced Díaz–González

**Affiliations:** 1 Servicio Canario de Salud, San Sebastián de la Gomera, Canary Islands, Spain; 2 Nursing Department, University of Las Palmas de Gran Canaria (ULPGC), Las Palmas de Gran Canaria, Canary Islands, Spain

**Keywords:** Tracheostomy, Tracheostomy patient, Tracheostomy tubes, Nursing care

## Abstract

**Introduction:**

Tracheostomy (TQT) is a procedure that maintains airway patency, and its effectiveness depends on the selection of appropriate equipment and specialized care. Knowledge gained during nursing and postgraduate training is essential to ensure safety and minimize risks in these patients. Identify the level of knowledge regarding TQT care among nurses at a hospital in the Canary Islands (Spain).

**Methodology:**

A cross-sectional study was conducted. The study population consisted of nurses from the hospital in the Canary Islands. The data collection instrument used was a self-completion questionnaire with 32 variables. The study was approved by the Ethics Committee 2023-502-1 and the Health Center Management.

**Results:**

The sample consisted of 38 participants (7.16%), which was not considered representative of the study population. The mean age was 39 years, with a predominance of women (81.58%), and a mean work experience of 15 years. 34.22% felt uncomfortable receiving care, and 47.37% sought help from colleagues. Critical issues, such as pre-feeding measures and decannulation practices, continue to create uncertainty among participants.

**Discussion/Conclusion:**

The findings are consistent with those reported in the scientific literature, where the level of knowledge regarding TQT care is generally described as moderate. The active involvement of healthcare institutions in continuous training is essential as a key strategy to improve staff knowledge and core competencies. In this regard, the implementation of interactive educational methodologies supported by technological tools—such as simulations and virtual reality games—may be an effective approach to optimizing both learning and clinical practice.

## Introduction

Tracheostomy (TQT) is a surgical procedure that involves making an opening in the anterior wall of the trachea at the level of the vocal cords, either temporarily or permanently, to maintain a patent airway and allow inspired air to pass from the outside to the bronchial tree through a cannula [1–5].

This procedure can be performed using a traditional operating room technique [6] or percutaneously (Seldinger technique) [6–9]. The latter is notable for its shorter time and invasiveness, as well as the lower risk of infectious complications 7–9, but is contraindicated in children [6]. The wide variety of TQT cannulas allows for maintaining airway patency by generating adequate positive pressure in the tracheostomy patient (TP) [1, 3, 5, 10]. Therefore, the choice of this type of cannula will depend on the individual’s characteristics and needs, such as age, size, and shape of the airway, as well as the diagnosis and clinical indications for placement (e.g., mechanical ventilation or phonation valves) [6].

For the care and safety of TP, healthcare professionals need to understand both the parts that make up a TQT cannula [1, 3, 4, 11] (external cannula, inner cannula, stylet, fenestration orifice, cuff), and the materials [1, 3, 10, 12] with which these cannula are made (mechanical, silicone, and polyvinyl chloride). Regarding the presence or absence of a cuff, it is vital that the professional controls the time of inflation and deflation of the tracheostomy cuff, as well as the recommended pressures of 20–25 mmHg routinely controlled with a manometer [1, 3–5, 10].

Although TQT may present complications [1, 5, 6, 9, 12, 13], the extraction of secretions at the tracheal or bronchial level using a sterile probe allows to maintain the patency of the airway with the greatest effectiveness in addition to maintaining a humid environment to fluidify said secretions, especially in the presence of visible secretions in the airway or obstructions due to mucous plug [6, 12, 14]. The American Academy of Respiratory Care (AARC) recommends limiting according to the need, quantity, and state of the secretions and trying not to increase the risk of infection or irritation of the mucosa [3, 5, 14]. Suction pressure should not exceed 120 mmHg to reduce the risk of hypoxemia, bleeding, mechanical injury to mucosal membranes, and atelectasis [12, 14]. The duration should not exceed 15 s in adults and 5 s in children, while the total procedure should not exceed three minutes [12]. Regarding changes and decannulations in TQT, these will depend on the clinical needs and conditions of each patient [1, 12, 15], but without ignoring the manufacturer’s instructions [1, 12]. For these procedures, as well as for stoma care, cannula changes, and maintaining airway patency, nurses must have the necessary equipment [16], proper patient positioning [3, 4, 14], knowledge, and skills to manage it effectively, thus ensuring patient safety and peace of mind while mitigating professional fears regarding the procedure.

Studies have shown that caring for TQT patients can generate anxiety and insecurity among nurses. Perhaps the origin of these feelings lies in a lack of knowledge about cannulas and TQT procedures, such as complete cannula changes or secretion aspiration, which are the most common actions. To provide adequate care for TP, nurses must have training that includes the knowledge and simulated situations that allow them to acquire skills. Providing quality care generates safety for TP, avoiding airway obstruction, infections, and bleeding, among others. Identifying the knowledge that nurses have can expose the gaps that allow planning and implementing continuous training in the hospital center. The study aimed to assess hospital nurses’ knowledge of TQT care and to examine the association between sociodemographic variables and this knowledge.

## Methods

A cross-sectional study was conducted. The study population consisted of university graduates nursing working at a hospital in the province of Las Palmas, Canary Islands, Spain (HPLP). This population consisted of 530 nurses on staff. Based on the sampling equation [17], a sample of 223 nurses was estimated to be representative of the study population (95% confidence level, precision +/- 5 units, population percentage 50%). However, as will be seen in the results section, the expected sample size was not reached: only 38 participants.

The inclusion criteria included nurses who were active in the Hospitalization Unit, Emergency Department, Intensive Care Unit, and Operating Room in the HPLP. In addition, they voluntarily signed the consent document to participate, completed, and responded to all the items of the questionnaire. However, those who did not complete all the items of the questionnaire were excluded.

After an extensive search for a validated questionnaire that would allow the assessment of nurses’ knowledge about TQT care, it was not possible to identify any, therefore, it was decided to design an ad hoc questionnaire, where some of the questions were obtained from different documents that evaluated knowledge [1–3, 5–7, 9, 10, 13, 18–21] and the rest was carried out with the support of guides [8, 22, 23], protocols [4, 11, 12, 14, 15], and counseling from two nurses (K.J.C & C.P.B) trained in TQT. The resulting instrument comprises 32 closed-ended items organized into four dimensions: (1) sociodemographic characteristics; (2) basic concepts related to TQT; (3) TQT cannulas; and (4) TQT care.

Thirty-two variables were included, where the independent variables were: (1) Age (years); (2) Gender (female/male/non-binary); (3) Years of professional activity (years); (4) Frequency of currently providing care to TP (daily/3–4 times a week/1–2 times a week/none); (5) Providing care to TP before current placement (yes/no); (6) Degree of comfort in caring for TP (uncomfortable/comfortable/very comfortable); (7) Frequency of asking a nursing colleague to accompany them to perform cannula changes (very frequently/frequently/rarely/never); (8) Knowledge acquired during their degree on TQT (numerical scale from 1 to 10); (9) Have they received ongoing training on TQT (yes/no). Among the dependent variables (Table 1), 23 questions were found that helped identify the knowledge that nurses have about cannulas and TQT care.

**Table 1:** Questionnaire on nurses’ overestimation of their knowledge level

	Items
1	The difference between tracheostomy and cricothyroidotomy:
2	Common complications in patients with tracheostomy do NOT include:
3	What is the function of the tracheostomy tube?
4	Tubes are classified by function, except:
5	What material are tracheostomy cannula made of?
6	Tubes consist of the following parts, except:
7	What function does the inner tube of the tube have?
8	All cannulas have the same flexibility
9	Regarding the tracheostomy tracheostomy cuff, it is correct that:
10	The objective of oxygen therapy through the tracheostomy is:
11	When a patient has thick secretions, the humidification should be lowered:
12	For what purpose is a wet gauze placed on top of the tracheostomy cannula when oxygen therapy is being administered by a route other than that used in this patient profile?
13	When a patient has a tracheostomy, before eating, the following should not be done:
14	How long should the suction of secretions in the tracheostomy last?
15	When suctioning secretions in the tracheostomy patient, how much pressure should the vacuum outlet of the aspirator be at?
16	The limit of aspirations per attempt:
17	How often should the first complete change of the tracheostomy cannula be performed?
18	During the complete cannula change, it may happen that the stoma hole in the skin:
19	Among the care during cannula changes:
20	How is cannulation performed?
21	In the cleaning and disinfection care of the cannulas you usually use (you can check several options)
22	Before proceeding to decannulation, it is necessary:
23	During a work shift you are caring for a 59-year-old patient who suffered an accidental decannulation last night. The on-call doctor was called and replaced the cannula with some difficulty. The patient’s oxygen saturation was 90% when the cannula was out, but it did not improve much once it was replaced. When you assess the patient, he is asleep in bed. You notice that his breathing is harder than normal and that the cannula plate is not stuck to the skin. When you try to aspirate, you do not get any secretion, and you encounter resistance. What could be happening?

The interpretation of the questionnaire was carried out by giving each question 0 points, 1 point for partially correct answers, and 2 points for correct answers. Based on this scoring system, the total possible score ranged from 0 to 46 points. Considering with low knowledge (0–15), medium knowledge (16–30), and high knowledge (31–46).

### Data collection method

The questionnaire was distributed using the free electronic survey administration software Google Forms [24]. The main author contacted all the nursing supervisors of the hospitalization units and services to request collaboration in the dissemination of the digital link to the questionnaire in the staff of active nurses, without this supervisor being able to know who has participated. Potential participants received information before accessing the questionnaire, including concise explanations of the objectives, research method, and data use, as well as the Ethics Committee’s acceptance. Participants were able to complete an anonymous questionnaire on their knowledge of TQT care (with prior voluntary acceptance of participation; only then would they be able to access this document). Data collection took place between January and February 2024.

### Statistical analysis

Data analysis was performed by exporting the data from Google Forms to Excel 2023 and analyzing it using the free JASP [25] version 0.16.4 program (Apple Silicon, Netherlands). Statistical analysis was performed based on: (1) Quantitative variables were expressed as range, mean, and standard deviation; (2) Qualitative variables were expressed as percentages and absolute frequencies. For bivariate analysis, different statistical tests were used based on the type of variable and its behavior (normal/non-normal distribution; paired/unpaired): Student’s t-test, Mann-Whitney U test, Pearson’s χ2 test, and for qualitative variables, contingency Table (2 × 2), Chi-square test, and Phi coefficient or contingency coefficient (dichotomous only/dichotomous-polytomous). Due to the risk of false positives due to the number of independent comparisons, it was decided to apply FDR (False Discovery Rate) Benjamini-Hochberg as the most balanced method [26].

Regarding ethical considerations, authorization was requested from the Canary Islands Health Service (SCS) and the Las Palmas Province Ethics and Research Committee (CEIm) to conduct the study, receiving an affirmative response with authorization code 2023-502-1. Based on the project presented at the CEIm, all participants received the participant information sheet (PIS), informed consent (IC), data protection (DP), and revocation (RV) forms in a pre-session section before their acceptance to participate and access to the questionnaire.

## Results

After the data collection period, the study sample consisted of 38 participants (*n* = 38), which was considered unrepresentative of the study population, representing a participation rate of 7.16%.

### Sociodemographic variables

The mean age of the sample was 39 years, with a standard deviation (SD) of 9.66 and a range of 24.0–64.0. The mean years of professional activity among participants amounted to 15 years, with an SD of 10.74 and a range of 0.0–44.0. Qualitative sociodemographic variables are represented as absolute frequencies and percentages, as shown in Table 2.

**Table 2:** Descriptive analysis of qualitative sociodemographic variables: frequencies and percentages

	Frecuency	Percent
**Gender**		
Male	7	18.42
Female	31	81.58
**Continuing education received**		
Yes	9	23.68
No	29	76.32
**Frequency with which you care for TP per week**		
Never	24	63.16
Half of the days	11	28.95
¾ of the days	2	5.26
Every day	1	2.63
**Have you provided care to TP?**		
No	1	2.63
Yes	37	97.37
**Professional comfort level with TP care**		
Uncomfortable	13	34.22
Comfortable	19	50.0
Very comfortable	6	15.78
**Frequency with which the professional asks a colleague for help when changing the TQT cannula**		
Never	9	23.68
Rarely	18	47.37
Frequently	10	26.32
Very frequently	1	2.63

Finally, according to the study participants, the mean score for knowledge acquired during the program was 4.03, with an SD of 1.72 and a range of 1.0–7.0, based on a numerical scale of 0–10.

### Analysis of the level of knowledge regarding TP care

The mean knowledge of the sample was 22.07, SD 5.44, with a minimum score of 13 and a maximum of 33 points, indicating moderate knowledge about care in TQT. At the low level of knowledge was 15.78%, at the moderate level 81.57% and at the high level 2.65%. Based on the responses provided by the 38 participants (Table 3), 63.1% answered incorrectly regarding the difference between a TQT and cricothyroidotomy. Approximately 78.9% (*n* = 30) of participants agreed that the least common complication in TP is air embolism, followed by tracheal granuloma in 10.5% (*n* = 4), and circulating skin necrosis or TQT obstruction in 5.3% (*n* = 2). Likewise, 100% of participants clearly understood the functions of a TQT cannula (preventing tracheal closure, ventilating the patient, and allowing for the aspiration and expulsion of pulmonary secretions).

**Table 3:** Frequency and percentage of responses to questionnaire items (Dichotomous: correct and incorrect; polytomous: correct, partially correct, and Incorrect)

	Answer	Frecuency	Percent	Items	Answer	Frecuency	Percent
Item 1. The difference between tracheostomy and cricothyroidotomy	Correct Incorrect Valid	14 24 38	36.84 63.16	Item 13. When a patient has a tracheostomy, before eating, the following should not be done	Correct Incorrect Valid	21 17 38	55.26 44.74
Item 2. Common complications in patients with tracheostomy do NOT include	Correct Incorrect Valid	4 34 38	10.53 89.47	How long should the suction of secretions in the tracheostomy last?	Correct Incorrect Valid	11 27 38	28.95 71.05
Item 3. What is the function of the tracheostomy tube?	Correct Incorrect Valid	38 0 38	100 0	Item 15. When suctioning secretions in the tracheostomy patient, how much pressure should the vacuum outlet of the aspirator be at?	Correct Incorrect Valid	12 26 38	31.58 68.42
Item 4. Tubes are classified by function, except:	Correct Incorrect Valid	13 25 38	34.2 65.78	Item 16. The limit of aspirations per attempt:	Correct Incorrect Valid	3 35 38	7.90 92.10
Item 5. What material are tracheostomy cannula made of?	Correct Incorrect Valid	10 28 38	26.32 73.68	How often should the first complete change of the tracheostomy cannula be performed?	Correct Incorrect Valid	12 26 38	31.58 68.43
Item 6. Tubes consist of the following parts, except:	Correct Incorrect Valid	30 8 38	78.95 21.05	Item 18. During the complete cannula change, it may happen that the stoma hole in the skin:	Correct Incorrect Valid	34 4 38	89.47 10.53
Item 7. What function does the inner tube of the tube have?	Correct Incorrect Valid	9 29 38	23.68 76.32	Item 19. Among the care during cannula changes:	Correct Incorrect Valid	4 34 38	10.53 89.47
Item 8. All cannulas have the same flexibility	Correct Incorrect Valid	37 1 38	97.37 2.63	Item 20. How is cannulation performed?	Correct Incorrect Valid	13 25 38	34.21 65.79
Regarding the tracheostomy tracheostomy cuff, it is correct that:	Correct Incorrect Valid	18 20 38	47.37 52.63	In the cleaning and disinfection care of the cannulas you usually use (you can check several options)	Correct Parcial correct Incorrect Valid	0 36 2 38	0.00 94.74 5.26
Item 10. The objective of oxygen therapy through the tracheostomy is:	Correct Incorrect Valid	19 19 38	50.00 50.00	Item 22. Before proceeding to decannulation, it is necessary:	Correct Incorrect Valid	33 5 38	86.84 13.16
Item 11. When a patient has thick secretions, the humidification should be lowered	Correct Incorrect Valid	36 2 38	94.74 5.26	Item 23. During a work shift you are caring for a 59-year-old patient who suffered an accidental decannulation last night…	Correct Incorrect Valid	21 17 38	55.26 44.74
Item 12. For what purpose is a wet gauze placed on top of the tracheostomy cannula when oxygen therapy is being administered by a route other than that used in this patient profile?	Correct Incorrect Valid	11 27 38	28.95 71.05				

Concerning the classifications of cannula functions, 31.6% (*n* = 13) of participants considered that composition was not a classification, while 13.2% (*n* = 5) did not consider cuff or diameter, and 7.9% (*n* = 3) did not consider fenestration. The remaining 34.2% (*n* = 13) identified all of the above classifications to be functions of the TQT cannula. Regarding the materials used in TQT cannula, 23.9% of participants (*n* = 9) highlighted polyvinyl, silicone, or metal, and polypropylene, silicone, or metal, compared to 21.2% (*n* = 12) who reflected polyethylene, silicone, or metal. When asked about the components of TQT cannula, 78.9% of participants (*n* = 30) stated that the midline cannula is not part of the TQT cannula, compared to 7.9% (*n* = 3) who pointed to the external or mother cannula, while 10.5% (*n* = 4) referred to the stylet or guidewire, and 2.6% (*n* = 1) to the inner cannala or endocannula. Likewise, 97.4% of respondents (*n* = 37) considered that cannulas have different flexibilities, compared to 2.6% (*n* = 1) who considered the opposite. Regarding the inner cannula’s specific functions, 23.7% (*n* = 9) stated that it only ensures tracheal patency, while 2.6% (*n* = 1) believed that it communicates with the outside of the trachea to prevent the closure of the TP or ensure the correct position of the mother cannula. In contrast, 71.1% of participants (*n* = 27) believed that both tracheal patency and communication with the outside of the trachea are specific functions of the inner cannula.

Regarding the question about the goals of oxygen therapy, 34.2% of respondents (*n* = 13) indicated only the goal of preventing or reversing hypoxemia in the TP, while the remaining 15.8% indicated that all answers were correct (preventing hypoxemia, maintaining high-flow oxygen, and humidifying mucous membranes to prevent dryness). Similarly, 50% (*n* = 19) answered that the goals were preventing hypoxemia and humidifying mucous membranes to prevent dryness. Regarding the question “In a patient with thick secretions, humidification should be reduced,” 94.7% of participants (*n* = 36) answered that the premise is false, as opposed to 5.3% (*n* = 2) who believed it to be true. Regarding what should not be done before eating in a TP, 53.2% (*n* = 21) indicate that the cuff should not be deflated, while 21.1% (*n* = 8) believe that the cuff should not be inflated, and 23.7% (*n* = 9) do not know what to do. Likewise, about question 18 regarding changing the complete cannula and the stoma orifice, 5.3% (*n* = 2) answered that the cannula should not match the tracheostoma or that trauma should be generated by friction in the peristomal area, in contrast to 89.5% (*n* = 34) who stated that both answers were correct. Regarding decannulation in a TP, 7.9% (*n* = 3) believe that it will occur depending on the prescription time and/or patient tolerance, while 5.3% (*n* = 2) believe that elective packing should be performed in the TQT, and 88.8% (*n* = 33) affirm that both answers are true. Regarding the clinical case presented in item 23, it stands out favorably that more than 55% of the sample selected the correct action.

### Relationship between sociodemographic variables and knowledge

For quantitative variables (age and years of professional activity), the Kolmogorov-Smirnov (KS) test was performed for normality. Parametric statistics showed a p-value of KS 0.069 (*p* > 0.05) for the age variable, with a normal distribution. The years of professional activity showed a KS of 0.019 (*p* < 0.05) with a lack of normal distribution, requiring nonparametric statistics. To determine a relationship between the sample’s sociodemographic variables and knowledge of TQT care, the following statistical tests were performed:

For the relationship between age (normal distribution – parametric statistics) and the professionals’ responses (correct and incorrect, unpaired dichotomous), a bivariate analysis was used using the Student’s t-test (Table 4). In most cases, no statistically significant differences were observed, with *p* > 0.05 across all comparisons, except for items 19 (during cannula changes) and 22 (needs before proceeding to decannulation). However, neither of these items retained statistical significance after applying the FDR correction.

**Table 4:** Student’s t-Test: comparison of age means based on correct/incorrect responses for each questionnaire item

Items del cuestionario	T	df	*P*	*P* (FDR)
Age-Item (1) The difference between tracheostomy and cricothyroidotomy:	0.171	36	0.865	0.891
Age-Item (2) Common complications in patients with tracheostomy do NOT include:	0.490	36	0.627	0.865
Age-Item (3) What is the function of the tracheostomy tube?	NaN	-	-	-
Age-Item (4) Tubes are classified by function, except:	0.372	36	0.712	0.865
Age-Item (5) What material are tracheostomy cannula made of?	0.859	36	0.396	0.756
Age-Item (6) Tubes consist of the following parts, except:	–1.157	36	0.255	0.543
Age-Item (7) What function does the inner tube of the tube have?	1.575	36	0.124	0.396
Age-Item (8) All cannulas have the same flexibility	NaN	-	-	-
Age-Item (9) Regarding the tracheostomy tracheostomy cuff, it is correct that:	0.633	36	0.531	0.842
Age-Item (10) The objective of oxygen therapy through the tracheostomy is:	–0.180	36	0.856	0.891
Age-Item 11. When a patient has thick secretions, the humidification should be lowered:	1.625	36	0.113	0.396
Age-Item 12. For what purpose is a wet gauze placed on top of the tracheostomy cannula when oxygen therapy is being administered by a route other than that used in this patient profile?	–0.138	36	0.891	0.891
Age-Item 13. When a patient has a tracheostomy, before eating, the following should not be done:	0.332	36	0.742	0.865
Age-Item 14. How long should the suction of secretions in the tracheostomy last?	1.693	36	0.099	0.396
Age-Item 15. When suctioning secretions in the tracheostomy patient, how much pressure should the vacuum outlet of the aspirator be at?	0.654	36	0.517	0.842
Age-Item 16. The limit of aspirations per attempt:	1.625	36	0.113	0.396
Age-Item 17. How often should the first complete change of the tracheostomy cannula be performed?	0.334	36	0.741	0.865
Age-Item 18. During the complete cannula change, it may happen that the stoma hole in the skin:	–1.148	36	0.259	0.543
Age-Item 19. Among the care during cannula changes:	2.422	36	0.021*	0.286
Age-Item 20. How is cannulation performed?	0.337	36	0.738	0.865
Age-Item 21. In the cleaning and disinfection care of the cannulas you usually use (you can check several options)	1.414	36	0.166	0.446
Age-Item 22. Before proceeding to decannulation, it is necessary:	–2.608	36	0.013*	0.286
Age-Item 23. During a work shift you are caring for a 59-year-old patient who suffered an accidental decannulation last night….	1.566	36	0.126	0.396

NaN Number of observation is < 2

*p < 0.05

False discovery rate (FDR) adjustment applied to original p-values (Benjamini–Hochberg Method)

The relationship between the participants’ years of professional experience and their knowledge of tracheostomy care (TQT) was analyzed using a bivariate Mann–Whitney U test (Table 5). A statistically significant difference was found only for item 22 (preparation before decannulation), with *p* = 0.014 (*p* < 0.05); however, this significance was lost after applying the false discovery rate (FDR) correction.

**Table 5:** Mann–Whitney U test: relationship between years of experience (YE) and responses to dichotomous and polytomous questionnaire items

Questionnaire Items	W	df	*P*	*P* (FDR)
YE-Item (1) The difference between tracheostomy and cricothyroidotomy:	181.000		0.705	0.799
YE-Item (2) Common complications in patients with tracheostomy do NOT include:	66.000		0.943	0.990
YE-Item (3) What is the function of the tracheostomy tube?	NaN		-	-
YE-Item (4) Tubes are classified by function, except:	150.500		0.723	0.799
YE-Item (5) What material are tracheostomy cannula made of?	175.000		0.242	0.651
YE-Item (6) Tubes consist of the following parts, except:	97.000		0.420	0.735
YE-Item (7) What function does the inner tube of the tube have?	162.500		0.279	0.651
YE-Item (8) All cannulas have the same flexibility	NaN		-	-
YE-Item (9) Regarding the tracheostomy tracheostomy cuff, it is correct that:	223.00		0.213	0.651
YE-Item (10) The objective of oxygen therapy through the tracheostomy is:	181.000		1.000	1.000
YE-Item 11. When a patient has thick secretions, the humidification should be lowered:	65.500		0.058	0.536
YE-Item 12. For what purpose is a wet gauze placed on top of the tracheostomy cannula when oxygen therapy is being administered by a route other than that used in this patient profile?	130.500		0.573	0.799
YE-Item 13. When a patient has a tracheostomy, before eating, the following should not be done:	196.000		0.617	0.799
YE-Item 14. How long should the suction of secretions in the tracheostomy last?	176.500		0.375	0.716
YE-Item 15. When suctioning secretions in the tracheostomy patient, how much pressure should the vacuum outlet of the aspirator be at?	192.500		0.257	0.651
YE-Item 16. The limit of aspirations per attempt:	60.500		0.684	0.799
YE-Item 17. How often should the first complete change of the tracheostomy cannula be performed?	172.500		0.615	0.799
YE-Item 18. During the complete cannula change, it may happen that the stoma hole in the skin:	47.000		0.329	0.691
YE-Item 19. Among the care during cannula changes:	91.500		0.273	0.651
YE-Item 20. How is cannulation performed?	180.500		0.590	0.799
YE-Item 21. In the cleaning and disinfection care of the cannulas you usually use (you can check several options)	61.500		0.102	0.536
YE-Item 22. Before proceeding to decannulation, it is necessary:	25.000		0.014*	0.294
YE-Item 23. During a work shift you are caring for a 59-year-old patient who suffered an accidental decannulation last night.	236.500		0.091	0.536

NaN Number of observation is < 2

*p < 0.05

False discovery rate (FDR) adjustment applied to original p-Values (Benjamini–Hochberg Method)

In search of a relationship between gender (dichotomous) and the sample’s knowledge about care in TQT, it was explored through the bivariate analysis of Chi-Squared (previously 2 × 2 contingency tables), and the Phi coefficient (PC) was included (Table 6), only a significant relationship was found between gender and how to perform the cannulation procedure (item 20), *p* 0.022 (*p* < 0.05) which disappears when FDR’s correction is performed. To understand the nature of this relationship, the frequencies observed in a contingency table were examined (Table 7), where the responses between men and women were: women with incorrect answers 23/31 and correct 8/31, compared to men 2/7 vs. 5/7, with men showing a higher proportion of correct answers. In the same table, continuous training on TQT care and knowledge, significant differences were only found with item 9 (knowledge about the tracheostomy cannula cuff), item 19 (cannula change procedure), item 21 (In the cleaning and disinfection care of the cannulas you usually use (you can check several options), and item 22 (needs before proceeding to decannulation) where *p* < 0.05, but again, these significant differences disappear when applying FDR.

**Table 6:** Chi-Square test and phi coefficient (PC) results from contingency table analysis for gender, receiving continuing education (CE) on TQT, and dichotomous item responses (Correct/Incorrect)

Gender-Items		Value	df	*P*	PC	*P* (FDR)	CE-Items		Value	df	*P*	PC	*P* (FDR)
Gender –Item (1) The difference between tracheostomy and cricothyroidotomy:	X^2^ *N*	0.133 38	1	0.715	–0.059	0.927	CE –Item 1	X^2^ *N*	3.356 38	1	0.067	0.297	0.281
Gender –Item (2) Common complications in patients with tracheostomy do NOT include:	X^2^ *N*	2.937 38	1	0.085	–0.269	0.623	CE –Item 2	X^2^ *N*	1.387 38	1	0.239	0.191	0.478
Gender –Item (3) What is the function of the tracheostomy tube?	X^2^ *N*	- -	0	-	-	-	CE –Item 3	X^2^ *N*	- -	0	-	-	
Gender –Item (4) Tubes are classified by function, except:	X^2^ *N*	0.121 38	1	0.728	0.056	0.941	CE –Item 4	X^2^ *N*	2.796 38	1	0.095	0.271	0.205
Gender –Item (5) What material are tracheostomy cannula made of?	X^2^ *N*	0.023 38	1	0.881	–0.024	0.969	CE –Item 5	X^2^ *N*	0.300 38	1	0.584	–0.089	0.720
Gender –Item (6) Tubes consist of the following parts, except:	X^2^ *N*	0.292 38	1	0.589	0.088	0.844	CE–Item 6	X^2^ *N*	0.010 38	1	0.922	0.016	0.223
Gender –Item (7) What function does the inner tube of the tube have?	X^2^ *N*	1.745 38	1	0.186	–0.214	0.715	CE–Item 7	X^2^ *N*	2.812 38	1	0.094	–0.272	0.207
Gender –Item (8) All cannulas have the same flexibility	X^2^ *N*	0.232 38	1	0.630	–0.078	0.866	CE–Item 8	X^2^ *N*	3.309 38	1	0.069	0.295	0.217
Gender –Item (9) Regarding the tracheostomy tracheostomy cuff, it is correct that:	X^2^ *N*	0.070 38	1	0.791	0.043	0.963	CE–Item 9	X^2^ *N*	4.374 38	1	0.036^*^	–0.339	0.252
Gender –Item (10) The objective of oxygen therapy through the tracheostomy is:	X^2^ *N*	1.576 38	1	0.209	0.204	0.640	CE–Item 10	X^2^ *N*	0.146 38	1	0.703	–0.062	0.819
Gender –Item 11. When a patient has thick secretions, the humidification should be lowered:	X^2^ *N*	0.477 38	1	0.490	–0.112	0.806	CE–Item 11	X^2^ *N*	0.809 38	1	0.368	0.146	0.598
Gender –Item 12. For what purpose is a wet gauze placed on top of the tracheostomy cannula when oxygen therapy is being administered by a route other than that used in this patient profile?	X^2^ *N*	5.8 × 10^− 4^ 38	1	0.981	0.004	0.969	CE–Item 12	X^2^ *N*	1.377 38	1	0.241	–0.190	0.478
Gender –Item 13. When a patient has a tracheostomy, before eating, the following should not be done:	X^2^ *N*	3.218 38	1	0.073	–0.291	0.803	CE–Item 13	X^2^ *N*	0.558 38	1	0.455	0.121	0.590
Gender –Item 14. How long should the suction of secretions in the tracheostomy last?	X^2^ *N*	0.897 38	1	0.344	0.154	0.688	CE–Item 14	X^2^ *N*	0.110 38	1	0.740	–0.054	0.819
Gender –Item 15. When suctioning secretions in the tracheostomy patient, how much pressure should the vacuum outlet of the aspirator be at?	X^2^ *N*	0.036 38	1	0.850	0.031	0.987	CE–Item 15	X^2^ *N*	0.903 38	1	0.342	–0.154	0.598
Gender –Item 16. The limit of aspirations per attempt:	X^2^ *N*	0.735 38	1	0.391	0.139	0.717	CE–Item 16	X^2^ *N*	3.329 38	1	0.068	–0.296	0.238
Gender –Item 17. How often should the first complete change of the tracheostomy cannula be performed?	X^2^ *N*	0.036 38	1	0.850	0.031	0.925	CE–Item 17	X^2^ *N*	0.017 38	1	0.897	–0.021	0.942
Gender –Item 18. During the complete cannula change, it may happen that the stoma hole in the skin:	X^2^ *N*	0.129 38	1	0.720	0.058	0.990	CE–Item 18	X^2^ *N*	0.004 38	1	0.948	0.011	0.948
Gender –Item 19. Among the care during cannula changes:	X^2^ *N*	1.009 38	1	0.315	0.163	0.693	CE–Item 19	X^2^ *N*	6.513 38	1	0.011*	–0.414	0.116
Gender –Item 20. How is cannulation performed?	X^2^ *N*	5.281 38	1	0.022*	–0.373	0.484	CE–Item 20	X^2^ *N*	0.753 38	1	0.386	0.141	0.579
Gender –Item 21. In the cleaning and disinfection care of the cannulas you usually use (you can check several options)	X^2^ *N*	0.477 38	1	0.490	–0.112	0.771	CE–Item 21	X^2^ *N*	6.802 38	1	0.009*	0.423	0.189
Gender –Item 22. Before proceeding to decannulation, it is necessary:	X^2^ *N*	1.300 38	1	0.254	–0.185	0.627	CE–Item 22	X^2^ *N*	4.201 38	1	0.040*	0.332	0.210
Gender –Item 23. During a work shift you are caring for a 59-year-old patient who suffered an accidental decannulation last night…	X^2^ *N*	0.012 38	1	0.912	–0.018	0.957	CE –Item 23	X^2^ *N*	0.620 38	1	0.431	–0.128	0.603

*p < 0.05

False discovery rate (FDR) adjustment applied to original p-Values (Benjamini-Hochberg Method)

**Table 7:** Contingency table analysis between gender and type of response to item 20: how to perform cannulation

Gender	Correct	Incorrect	Total
Female	8	23	31
Male	5	2	7
Total	13	25	38

Regarding the frequency with which the professional pays attention to TP, significant differences were found related to knowledge about “reducing humidification in the presence of thick secretions” (item 11), *p* < 0.001 — a difference that remained significant after applying FDR correction (*p* = 0.011; *p* < 0.05) — and was associated with a moderate contingency coefficient (CC = 0.585).

For item 21, “In the cleaning and disinfection care of the cannulas you usually use,” significant differences remained after applying FDR correction (*p* = 0.011; *p* < 0.05), as also observed in the previous item. In contrast, for item 19, “the first complete change of the tracheostomy tube,” although the initial difference was statistically significant (*p* = 0.031; *p* < 0.05), this significance disappeared after FDR adjustment (Table 8).

**Table 8:** Chi-Square test and contingency coefficient (CC): frequency of professional care for the tracheotomy patient (FPCTP) (Ordinal) and questionnaire responses

		Value	df	*P*	CC (Phi /Cramer´s V^∇^)	*P* (FDR)
FPCTP –Item (1) The difference between tracheostomy and cricothyroidotomy:	X^2^ *N*	1.405 38	3	0.704	0.189	0.704
FPCTP –Item (2) Common complications in patients with tracheostomy do NOT include:	X^2^ *N*	4.659 38	3	0.199	0.330	0.508
FPCTP –Item (3) What is the function of the tracheostomy tube?	X^2^ *N*	NaN -	-	-	-	-
FPCTP –Item (4) Tubes are classified by function, except:	X^2^ *N*	1.092 38	3	0.779	0.867	0.793
FPCTP –Item (5) What material are tracheostomy cannula made of?	X^2^ *N*	5.162 38	3	0.160	0.346	0.508
FPCTP –Item (6) Tubes consist of the following parts, except:	X^2^ *N*	1.330 38	3	0.722	0.184	0.724
FPCTP –Item (7) What function does the inner tube of the tube have?	X^2^ *N*	1.032 38	3	0.793	0.163	0.793
FPCTP –Item (8) All cannulas have the same flexibility	X^2^ *N*	2.521 38	3	0.472	0.249	0.508
FPCTP –Item (9) Regarding the tracheostomy tracheostomy cuff, it is correct that:	X^2^ *N*	1.156 38	3	0.764	0.172	0.764
FPCTP –Item (10) The objective of oxygen therapy through the tracheostomy is:	X^2^ *N*	2.485 38	3	0.478	0.248	0.508
FPCTP –Item 11. When a patient has thick secretions, the humidification should be lowered:	X^2^ *N*	19.768 38	3	< 0.001**	0.585	0.011*
FPCTP –Item 12. For what purpose is a wet gauze placed on top of the tracheostomy cannula when oxygen therapy is being administered by a route other than that used in this patient profile?	X^2^ *N*	1.517 38	3	0.678	0.196	0.704
FPCTP –Item 13. When a patient has a tracheostomy, before eating, the following should not be done:	X^2^ *N*	4.109 38	3	0.250	0.312	0.508
FPCTP –Item 14. How long should the suction of secretions in the tracheostomy last?	X^2^ *N*	3.064 38	3	0.382	0.273	0.508
FPCTP –Item 15. When suctioning secretions in the tracheostomy patient, how much pressure should the vacuum outlet of the aspirator be at?	X^2^ *N*	3.271 38	3	0.352	0.282	0.508
FPCTP –Item 16. The limit of aspirations per attempt:	X^2^ *N*	2.317 38	3	0.509	0.240	0.509
FPCTP –Item 17. How often should the first complete change of the tracheostomy cannula be performed?	X^2^ *N*	0.957 38	3	0.812	0.157	0.812
FPCTP –Item 18. During the complete cannula change, it may happen that the stoma hole in the skin:	X^2^ *N*	3.573 38	3	0.311	0.293	0.508
FPCTP –Item 19. Among the care during cannula changes:	X^2^ *N*	8.882 38	3	0.031*	0.435	0.227
FPCTP –Item 20. How is cannulation performed?	X^2^ *N*	1.698 38	3	0.637	0.207	0.686
FPCTP –Item 21. In the cleaning and disinfection care of the cannulas you usually use (you can check several options)	X^2^ *N*	19.768 38	3	< 0.001**	0.263^∇^	0.011*
FPCTP –Item 22. Before proceeding to decannulation, it is necessary:	X^2^ *N*	4.453 38	3	0.217	0.324	0.508
FPCTP –Item 23. During a work shift you are caring for a 59-year-old patient who suffered an accidental decannulation last night.	X^2^ *N*	3.052 38	3	0.384	0.273	0.508

*p < 0.05. **p < 0.001. ^∇^Cramer´s V coefficiente

False discovery rate (FDR) adjustment applied to original p-Values (Benjamini–Hochberg Method)

Finally, having received ongoing training was not significantly related to the level of comfort in providing care to the TP (Table 9) (*p* 0.122; *p* > 0.05). The same was true for ongoing training and the frequency with which the provider requested assistance from a colleague when changing the TQT cannula, *p* 0.075; *p* > 0.05.

**Table 9:** Chi-Square test and contingency coefficient (CC) analysis of continuous training received (Yes/No), nurse comfort when providing care to the tracheostomy patient (TP), and occasions when the professional requests help

	Value	Df	*p*	CC
**Continuing education received & Nurse comfort when providing care to the TP**				
X^2^	4.211	2	0.122	0.316
*N*	38			
Continuing education received & Occasions in which the nurse requests help from other colleagues				
X^2^	6.895	3	0.075	0.392
*N*	38			

## Discussion

It is important to remember that the sample was not considered representative of the study population. The mean age of the study participants was 39 years, much higher than that found in other lines of research (mean age between 20 and 28 years) [27]. Regarding gender, the sample consisted predominantly of women (81.58%), consistent with findings from other studies (88%) [27], highlighting that the nursing profession remains primarily female-dominated. Regarding the participants’ years of professional experience, the present study shows an average of 15 years of experience, compared to other studies with professionals between 1 and 4 years of work experience [27, 28].

### Continuing education

This study shows the high percentage of participants who have not received continuing education on TQT (76%). However, 97% report having provided care to TP at some point in their professional career. These data possibly indicate that care delivery may not reach excellence. A study conducted at the IDC Salud Hospital Universitari Sagrat Cor [29] identified that 89.13% of respondents would like to receive more training, with those who had received training reporting improved skills and knowledge, and 78.28% declaring that they had put into practice aspects learned during their training [29]. Regarding the knowledge acquired during university studies, a study conducted in Havana [28] identified insufficient knowledge about TQT, stating that 86.6% had low preparation. Furthermore, lack of training could be one of the causes of the lack of comfort in asking for help from peers. but it would be convenient to include in future studies based on previous research where it has been identified that confidence, security and comfort in professional activity is related to knowledge in the subject, where some authors highlight the importance of health institutions offering specific training programs since such initiatives would have a positive effect on nursing skills, patient care and the motivation and commitment of nurses [30–32].

### Most notable knowledge in the sample

Considering that the sample presents a moderate level of knowledge (mean 22.07) on a possible scale of 0–46, with 81.57% of participants falling within the moderate range (16–30). This level is similar to that found in other studies [33, 34]. Gaterega et al. [34] showed that most of the nurses interviewed (71%) had a moderate level of knowledge. However, when the practical level was evaluated, a greater number exhibited a low level (97.5%).

Due to the limited participation in the study population (*n* = 38, studies have been identified that show similarities. Mosalli et al. [35] obtained a sample of 43 nurses, and the intervention control groups from Yousif et al. [36] consisted of 45 participants.

Focusing on questions related to TQT knowledge, these questions will be addressed based on the percentage of correct answers. As noted in the results section, participants showed the greatest knowledge (> 75% correct answers) in the following areas: (a) the functions, parts, and flexibility of the cannulas. 100% correctly selected the cannula functions, and approximately 98% of nurses identified that the flexibility of TQT cannulas will depend on the materials used; (b) humidification in the presence of secretions, where 94.7% of participants stated that increasing humidification helps prevent mucus plugging. According to the scientific literature, excessive or insufficient humidification promotes secretion retention, the development of atelectasis, and hypoxemia [5]. Thus, the study by Sztrymf et al. [37] demonstrates that the use of high-flow oxygen therapy allows better management of respiratory secretions in the TP, thereby increasing oxygenation levels and reducing hypoxemia. Furthermore, it is striking that 68.4% of the total sample lacked knowledge about the use of wet gauze during oxygen administration in a TQT cannula. Other relevant knowledge gaps include: (c) situations that may arise when changing cannulas, and (d) preparation before changing the cannula.

Regarding responses with less than 50% correct answers, it is worth noting that less than half of the nurses correctly answered the differences between TQT and cricothyroidotomy. 78.9% incorrectly selected that the midline cannula is not a component of the cannula. Regarding the composition and components of TQT cannula, the low level of knowledge among respondents stands out; 35% stated that fenestration, diameter, and cuff are classifications of cannula, and 23.9% highlighted polyvinyl, silicone, or metal as materials. In contrast, a lack of knowledge was observed regarding the function of the inner cannula, with only 23.7% of respondents identifying the correct answer.

A mere 10.5% selected tracheal granuloma as the least common complication among the different options. According to the scientific literature, tracheal granuloma is considered a late complication and, at the same time, the most common in this classification, with an incidence ranging from 4 to 80% [38].

Regarding knowledge of cuff, a discrepancy is observed between the available options; 21.1% indicated that the correct volume was between 25 and 30 cm H_2_O, and 26.3% between 20 and 25 cm H_2_O. Therefore, 46% provided the correct answer. It is necessary to highlight the importance of the question about possible bronchoaspiration as an excess of pressure that damages the area producing necrosis, due to the presence of discrepancy observed among the respondents, although it is necessary to emphasize that it may be due to the different ranges of normality found in the scientific literature, with values ​​between 20 and 25 cm H_2_O [3, 5] or between 20 and 30 cm H_2_O [4], generating doubts among health professionals regarding the adequate volume.

Regarding the aspiration of secretions, a lack of knowledge of the respondents in aspects such as vacuum intake pressure, time, and limit in this procedure is observed, with percentages of incorrect responses of 44.7%, 65.8%, and 68.4% respectively. According to Uceda et al. [39] the professionals in the critical patient’s room of the emergency service of the Guillermo Almenar Irigoyen National Hospital, presented a 60% medium-low level of compliance in the aspiration of secretions either due to lack of time or work overload of the staff, although it was oriented to evaluate the skills and abilities, the knowledge was not evaluated directly, the possible lack of these could be one of the factors that influenced the negative results, for this reason, the absence of several steps can lead to complications such as hypoxia or injuries to the tracheal mucosa. Focusing on the prevention of hypoxia, the previously mentioned study [39] shows that 55% do not hyperoxygenate the patient before the procedure, and 75% of these also fail to consider the duration of suctioning. Undoubtedly, another moment of great importance in the procedure is the precautions that the professional must take before the TP eats; more than 44% of those surveyed did not consider deflating the tracheostomy cuff as a procedure to avoid before oral feeding. Considering the scientific literature, the cuff in a TQT must be inflated before, during, and after food intake, since otherwise the risk of bronchoaspiration would increase and, therefore, the risk of tamponade and cardiorespiratory arrest would increase [40].

Regarding the change of TQT cannula, it is worth highlighting discrepancies among the participants, since approximately 1 in 4 nurses perform the first cannula change between the first and second week, while in the complete change of cannula and the stomal orifice, 89.5% of the participants consider that trauma can occur, and the cannula cannot match. As reported in the study by Peter et al. [41], the implementation of educational programs, simulations, and demonstrations on tracheostomy care contributes to improving the confidence, knowledge, and competence of health professionals with cannula changes in TP. Along these lines, Brenner et al. [42] the use of interactive, competency-based education, research on clinical practice, and improvement in the quality of care generate stronger evidence, as well as greater effectiveness and refinement in health care practices aimed at TP. Biyik et al. [43] shows that the use of new technologies based on virtual reality games constitutes a support for training, favoring the development of knowledge and skills in both health professionals and nursing students.

Previous studies show that the first cannula change should be performed between 7 and 14 days after TQT placement [1, 5, 12] and, depending on whether the TQT is surgical or percutaneous, it is recommended from the 5th or 10th day [3]. Professionals in general show some concern about the first TQT cannula change performed on the TP, which may be due to the incidence of complications that usually occur. Jalil et al. [44] showed in his study that 6% of the changes of this device in the TP presented complications, among which peristomal bleeding (47.37%) and a failed first attempt (34.21%) stand out, possibly related to an increase or decrease in the internal stomal diameter, these complications being up to 5 times more frequent.

Returning to the results obtained in the study in the HPLP, the cleaning, and use of disinfectants in TQT cannulas, none of the respondents selected the three correct answers (soap and water, glutaraldehyde, hydrogen peroxide), but considering the choice of some of the correct options, 94% marked at least one or two correct, compared to 5% who did not select any correct. However, a study by Fernández García [9] indicated that the cure performed with PHMB or polyhexamethylene biguanide showed a slight tendency to decrease the risk of infections performed with povidone iodine. The use of this type of disinfectant will depend largely on the protocols that exist in each care unit [16].

Regarding cannula care, it is worth noting that only 10.5% of respondents selected that, before cannula placement, the neck of the TP should be placed in hyperextension, and that for cannulation, only 34.2% of respondents referred to entering the cannula at 3 o’clock and turning it towards 6 o’clock (clockwise). Likewise, regarding decannulation, a large proportion of participants stated that it depends on patient tolerance and the use of scheduled tamponades (88.8%), while in the clinical case question, more than half of the nurses identified that they could be faced with a possible false pathway, and the cannula should be removed. Ribeiro et al. meta-analysis [45] points to the Malhadevan series with a 75% success rate in decannulated patients and a 6.5% success rate in cases requiring recannulation, as is the case in similar studies such as the one conducted at the Hospital Clinic of Barcelona [46] where decannulation success rates reached 72.72%. Finally, regarding care during cannulation and decannulation, the scientific literature indicates that the use of slight hyperextension of the patient’s neck, force-free cannulation, and recommendations for the first cannula change help reduce the risk of creating a false pathway in the TP [4, 12].

This study, like others [47], did not find a statistically significant relationship between the level of knowledge and variables such as age, educational level or work experience. However, Abu-Sahyoun et al. [33] did report significant differences, which could be explained, at least in part, by the larger sample size used in their research (*n* = 260), which would have increased the statistical power to detect associations.

### Limitations

It should be noted that the study had certain limitations that may have influenced the reported results. Among them, the very low participation rate could be attributed to various factors, such as the high care load of nursing staff, forgetting to participate in the stipulated time, the lack of interest or familiarity with the topic addressed, the possible mistrust or reluctance related to the confidentiality of the data, despite having guaranteed anonymity. This small sample limits the statistical power of the analyses carried out and is especially relevant in those categories in which only one participant is available, which prevents valid comparisons from being made and can generate distortions in the interpretation of the data due to the disproportionate weight of atypical observations. On the other hand, complications in finding validated questionnaires related to TQT care, as well as the difficulty in finding recent studies. All of this has been obstacles to achieving a representative sample among HPLP nurses, in addition to pointing out the difficulty in comparing results and having a more enriching discussion.

## Conclusions

This study revealed a low level of participation and a moderate level of knowledge among participants about the care of patients with tracheostomy, with an average of 48% correct answers. No significant differences were identified between the variables analyzed and the level of knowledge, except in the case of continuing education. Given the clinical relevance of these findings, it is proposed to review and strengthen nursing curricula for undergraduate students, as well as to promote continuing education programs within health institutions. In both cases, the incorporation of interactive educational methodologies based on competencies, together with technological resources such as simulations and virtual reality games, can favor the development of essential knowledge and skills. Strengthening these competencies in nurses is a key element in reducing complications during care and ensuring safe, high-quality care for TP.

## Data Availability

The datasets used and analyzed during this study are available from the corresponding author upon reasonable request.

## Abbreviations

CC: Contingency Coefficient
CE: Continuing Education
HPLP: Hospital of the Province of Las Palmas
PC: Phi Coefficient
PHMB: Polyhexamethylene biguanide
SD: Standard Deviation
TP: Tracheostomy Patients
TQT: Tracheostomy
